# Increasing Superficial Musculoaponeurotic System Durability and Retention in Facelift Surgery

**DOI:** 10.1093/asjof/ojaf126

**Published:** 2025-10-17

**Authors:** Vlad Tereshenko, Madison R Hussey, Michael C McCormack, William G Austen

## Abstract

Background: Facelift surgery remains the gold standard for facial rejuvenation, yet limitations such as suture pull-through failure, superficial musculoaponeurotic system (SMAS) laxity, and the need for secondary procedures persist. Although novel facelift techniques have emerged, none have effectively addressed the inherent biomechanical challenges. Photochemical tissue passivation (PTP) is a promising method to enhance tissue durability and improve facelift longevity by modifying the biomechanical properties of the SMAS itself. Objectives: In this study, the authors investigated whether PTP enhances the biomechanical properties of SMAS, strengthens tissue integrity, and improves suture retention strength, thereby reducing the likelihood of recurrent laxity and the need for revision facelifts. Methods: SMAS tissue was harvested from facelift patients (*n* = 7) and Lewis rats (*n* = 16). The harvested SMAS underwent treatment with PTP. After treatment, the biomechanical properties, including modulus of elasticity, maximum load, and suture pull-through resistance, were assessed. Additionally, an in vivo rat SMAS plication model was established, and tissue laxity was evaluated at 4 and 12 weeks postoperatively. Results: PTP-treated human SMAS demonstrated a 229% increase in suture pull-through resistance (*P* = .21) and significantly higher modulus of elasticity in both human and animal specimens (*P* = .011 and *P* = .013, respectively). In the in vivo rat model, the laxity of SMAS plication was significantly lower by 58% at 4 weeks (*P* = .005) and 54% at 12 weeks (*P* < .0001) compared with untreated controls. Conclusions: PTP significantly enhances the biomechanical strength of the SMAS, reduces postoperative laxity in a rat model, and improves suture retention capacity in human SMAS tissue. These effects indicate that PTP has the potential to substantially increase the durability of facelifts and reduce revision rates, positioning it as a valuable adjunct technique in facial rejuvenation surgery. Additional clinical studies are needed to confirm these findings.

**Level of Evidence:** 5 (Therapeutic)

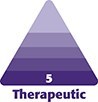

Rhytidectomy, or facelift surgery, is one of the most effective modalities for addressing facial aging by reducing wrinkles and sagging skin.^1^ Introduced over 100 years ago, facelift techniques have undergone numerous refinements, including deep-plane, superficial musculoaponeurotic system (SMAS) based, and minimally invasive approaches.^2^ Despite these advancements, facelift surgery still presents challenges, particularly in terms of recurrence of sagging, limited longevity of results, and the frequent need for secondary procedures.^3,4^ Although a broad spectrum of surgical techniques are available, no major disruptive innovations have fundamentally transformed rhytidectomy in recent decades.^1^

Initially, facelift techniques relied primarily on skin excision, but modern consensus emphasizes the importance of manipulating the SMAS.^5^ The SMAS is an essential anatomical structure in facial aesthetics, providing a durable and more natural-looking lift. Its biomechanical and anatomical properties have been extensively studied, with recent reports further elucidating its intricate structure and functional sophistication.^6,7^ Although SMAS-based techniques offer advantages over skin-only lifts, they are limited by suture failure, tissue fragility under tension, and a lack of long-term stability, often resulting in the recurrence of sagging and the need for revision procedures because of the SMAS failing to retain its position.^5^ Despite extensive refinements in surgical techniques, there have been minimal advancements in improving the intrinsic biomechanical properties of the SMAS itself.

Modifying the biomechanical properties of soft tissue is inherently challenging.^8^ Although regenerative technologies and structural enhancements have shown promise in bone tissue engineering, comparable advancements in soft tissue manipulation remain a major focus of research.^9,10^ One emerging modality, photochemical tissue passivation (PTP), has demonstrated potential for enhancing the mechanical integrity of biological tissues.^11-13^ PTP involves applying a photoactive dye to the tissue, followed by exposure to light at a specific wavelength, which facilitates covalent crosslinking of collagen molecules, thereby improving tissue durability and mechanical stability. This technique has been successfully applied in various biological tissues in animal models, with ongoing clinical trials exploring its clinical applications in other surgical fields.^11,14^

In this study, we aimed to enhance the durability of the SMAS and prevent laxity in a SMAS plication model. First, we assessed the effects of photochemical passivation on human SMAS tissue, demonstrating improved biomechanical properties and increased resistance to suture tear-through. Second, we established an in vivo SMAS plication model in rats, where PTP-treated SMAS exhibited enhanced tissue resilience and improved plication stability up to 3 months postoperatively.

## METHODS

### Human Specimen Enrollment

Seven participants were recruited for this study at Massachusetts General Hospital from January 2023 to December 2024, irrespective of sex, gender, race, or ethnicity. All participants underwent a primary facelift procedure. Inclusion criteria were age ≥18 years, undergoing a primary facelift, ability to provide informed consent, and fluency in English for consenting purposes. Exclusion criteria included pregnancy and inability to provide informed consent. All facelift procedures were performed by a fully trained plastic surgery attending (W.G.A.). During surgery, a 3 × 2 cm SMAS sample was harvested from the neck region at the level of paramedian platysma plication. Because this tissue is routinely resected for optimal neck lift outcomes, no additional tissue was harvested specifically for this study. Patient-specific demographic data included sex, date of birth, and age at the time of the first surgical procedure. Informed consent was obtained from all participants, and ethical approval was granted by the Massachusetts General Brigham IRB (protocol numbers: 2020P004060 and 2020P003555).

### Animals and Study Design

Sixteen male Lewis rats (aged 8-10 weeks, weighing ∼250 g) were utilized in this study. The rats were stratified into 3 experimental groups. Group A (*n* = 5) was utilized to analyze the effects of PTP on native SMAS at time zero. Group B (*n* = 4) was utilized to assess the effects of PTP on SMAS plication 1 month postsurgery, whereas Group C (*n* = 7) was utilized to evaluate the effects of PTP on SMAS plication 3 months postsurgery.

In each rat, 1 side was treated with PTP, whereas the contralateral side served as an internal control. The PTP treatment was performed in vivo in all groups. In Group A, SMAS tissue was harvested bilaterally immediately after the unilateral PTP treatment. In Groups B and C, PTP treatment was performed first, followed by bilateral SMAS plication. In Group B, the follow-up period was 4 weeks; in Group C, the follow-up lasted 12 weeks. At the designated follow-up points, dehiscence of the SMAS plication was measured, followed by SMAS tissue harvesting, biomechanical testing, and histological analysis using Masson's trichrome (MGT) staining.

Rats were housed under standardized conditions, with a 12-h light/dark cycle, and had ad libitum access to food and water. Preoperatively, all animals received subcutaneous injections of 0.05 mg/kg s.c. buprenorphine and 2 to 5 mg/kg s.c. of carprofen for analgesia. Carprofen (2-5 mg/kg s.c.) was given daily during the first 3 postoperative days. All housing and procedures were conducted in accordance with Institutional Animal Care and Use Committee (IACUC) guidelines, and ethical approval was obtained from the Massachusetts General Hospital IACUC.

### Surgery for superficial musculoaponeuroticsystem (SMAS) Plication in a Rodent Model

**Figure 1.**
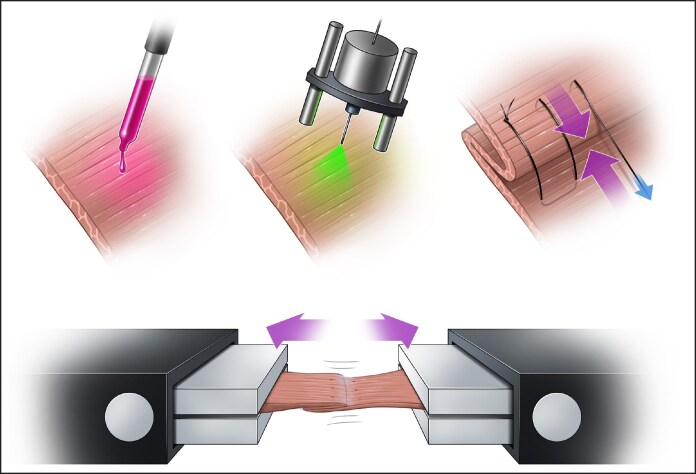
Study design in animal model. This schematic illustrates the workflow of the in vivo trial investigating the effects of PTP on SMAS plication. After bilateral facial dissection in the rat model, the superficial layer of the platysma was exposed. Rose Bengal dye was topically applied to the targeted area and excess dye was removed. The tissue was then irradiated with a continuous wave 532 nm KTP laser for 270 s to induce crosslinking. Following PTP treatment, the SMAS was plicated using interrupted sutures. Biomechanical analysis of the treated tissues was performed at 4 and 12 weeks postoperatively using uniaxial tensometry. PTP, photochemical tissue passivation; SMAS, superficial musculoaponeurotic system.

The experimental SMAS plication surgery was established in the rodent model following multiple modifications in pilot groups, ultimately resulting in a clinically translatable procedure (Figure 1). Anesthesia was induced by inhalation of 5% isoflurane and maintained at 1% to 3% through a nose cone. Preoperative analgesia was administered through subcutaneous injections of buprenorphine 0.05 and 2 to 5 mg/kg s.c. of carprofen.

Each rat was positioned in a lateral decubitus position on the corresponding side. A modified L-shaped incision was made, starting 5 mm ventral to the medial canthus and extending ∼2 cm caudally, terminating 1 to 2 mm below the intratragal notch. A second 2 cm incision was extended ventrally at a 90° angle. Using microinstruments under magnification, careful dissection of the skin flap was performed to ensure a clean separation from the underlying SMAS surface. After exposing a 3 × 3 cm area, surgical steps varied according to the experimental group.

In Group A, PTP treatment was administered unilaterally after SMAS exposure. Subsequently, a 1.5 × 0.5 cm SMAS strip was harvested from the treated side for biomechanical testing. On the contralateral side (internal control), the same procedure was performed, excluding PTP treatment, and a corresponding SMAS sample was collected.

In Groups B and C, PTP treatment was applied unilaterally following SMAS exposure, and SMAS plication was subsequently performed. The SMAS plication area (2 × 1.5 cm) was premarked to ensure reproducibility. To accurately measure SMAS plication dehiscence over time, two 5-0 Prolene (polypropylene, Ethicon, Johnson & Johnson, Somerville, NJ) marker threads were placed parallel to the ventral and dorsal marking lines. A 23G needle was utilized to insert the threads at the caudal end of the markings, guiding them through the tissue and exiting at the cranial end. The needle was removed, leaving the marker threads embedded within the SMAS to track plication stability.

**Figure 2.**
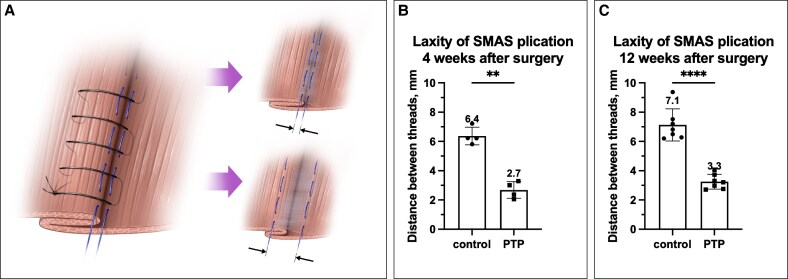
Evaluation of SMAS plication laxity in the animal model. (A) Schematic representation of SMAS plication and its assessment method. The distance between marking threads was measured using a caliper under magnification to quantify laxity. (B) Four weeks postsurgery (*n* = 4), SMAS plication laxity was significantly lower in the PTP-treated group compared with the control (2.7 ± 0.6 vs 6.4 ± 0.6 mm, paired *t*-test, *P* < .01). (C) Twelve weeks postsurgery (*n* = 7), the PTP-treated SMAS maintained improved structural integrity with reduced laxity compared with the control (3.3 ± 0.5 vs 7.1 ± 1.1 mm, paired *t*-test, *P* < .0001). **P < .01; ****P < .0001. PTP, photochemical tissue passivation; SMAS, superficial musculoaponeurotic system.

The plication was performed using a noninterrupted 5-0 Vicryl Plus (polyglactin 910, Ethicon, Johnson & Johnson) suture in a caudo-cranial direction, incorporating the 5-0 Prolene marker threads into the suture. Once SMAS plication was completed, skin closure was performed using interrupted subcutaneous 5-0 Vicryl Plus sutures, followed by a running baseball stitch with 5-0 Monocryl (poliglecaprone 25, Ethicon, Johnson & Johnson). Each rat was then repositioned in the contralateral lateral decubitus position, and an identical SMAS plication was performed on the opposite side, excluding PTP treatment. At 4 weeks (Group B) and 12 weeks (Group C) postsurgery, evaluations of plication dehiscence and tissue harvesting were performed. After raising the skin flap, the distance between the marker threads was measured at 5 equidistant points from caudal to cranial under a microsurgical microscope (Figure 2A). Following the measurements, a bilateral 2 × 2 cm SMAS sample was harvested for biomechanical testing and histological analysis.

### Photochemical Tissue Passivation

PTP is a well-established technique that has been applied to various tissue types in both small and large animal models. In this study, PTP was performed on SMAS tissue in both human and animal experiments. In the human trial, ex vivo PTP treatment was applied to SMAS tissue, whereas in the rat model, PTP was performed in vivo (Figure 1).

For PTP treatment, a sterile solution of 0.1% (w/v) Rose Bengal (RB) was prepared before surgery by dissolving solid RB (Sigma-Aldrich) in sterile normal saline (Baxter) and then passing the solution through a 0.22 μm filter (EMD Millipore, Burlington, MA). The dye was applied using a cotton-tip applicator. Any excess RB was absorbed with sterile gauze to ensure controlled application. The SMAS tissue was then illuminated at 532 nm from a continuous wave KTP laser (Laserscope Aura-i, San Jose, CA irradiance of 0.312 W/cm^2^) for 270 s.

### Biomechanical Testing

The biological effects of PTP were assessed through biomechanical testing in both human and animal trials. The mechanical properties of native SMAS were evaluated to determine the impact of PTP treatment, whereas sutured SMAS specimens were tested to assess tissue tearing resistance at the suture–tissue interface. The presence of a suture created a localized stress concentration where it entered and exited the tissue, simulating the suture-holding strength observed in SMAS plication procedures. Tissue failure at the suture–tissue interface, rather than across the entire specimen, indicates the tissue's ability to retain the suture before tearing.

SMAS specimens were standardized into rectangular samples measuring 5.0 mm in width and 2.0 mm in length, with a thickness of 2.0 mm, resulting in a cross-sectional area of 10.0 mm^2^ to ensure consistency across tests. All samples were kept hydrated in phosphate-buffered saline (PBS) until testing to prevent desiccation and alteration of biomechanical properties.

In the human trial, each patient sample was split into 2 strips: one undergoing PTP treatment and the other serving as a control. The first set of tests compared the biomechanical properties of native treated vs nontreated SMAS samples, whereas the second set evaluated suture retention strength. A 6-0 Vicryl suture was placed 2 mm from the edge at 1 end of the SMAS specimen, which was then fixated onto 1 clamp, whereas the opposite end was secured in the opposing clamp.

In the animal trial, the effects of PTP on native SMAS were tested immediately after treatment application. In Groups B and C, the effects of PTP-treated SMAS plication were assessed at 4 and 12 weeks postoperatively, respectively.

Tensile testing was conducted using a uniaxial mechanical testing system (300 Series Micro Test System Static Load Cell/10N) to measure applied force and an extensometer (Admet MTestQuattro) to record displacement. The tissue specimens were secured between 2 customized 3-dimensionally printed clamps, ensuring even force distribution across the tissue structure. Tests were performed at room temperature, with samples immersed in PBS solution. A displacement-controlled protocol was employed, in which specimens were stretched at a constant strain rate of 3 mm/min until mechanical failure. Force (N) and extension (mm) data were continuously recorded throughout the test.

Load-extension curves were generated from the recorded data to determine maximum load, modulus of elasticity, and, in human tissue, maximum stress. The maximum load was defined as the peak force the tissue could withstand before failure. At the same time, the modulus of elasticity was calculated from the linear region of the stress–strain curve to assess tissue stiffness. In human samples, maximum stress was also analyzed as a measure of the tissue's ability to withstand applied force relative to its cross-sectional area. These parameters were used to evaluate the biomechanical properties of SMAS tissue under tensile stress.

### Histology

For MGT staining, SMAS samples were fixed in 4% paraformaldehyde for up to 24 h before being rinsed with PBS for an additional 24 h. Following fixation and rinsing, the samples were embedded in paraffin to prepare for histological analysis.

The tissue samples were placed in tissue cassettes and subjected to dehydration using a microwave tissue processor (KOS, Milestone, Italy). The dehydration sequence included 35 min in 100% ethanol, followed by 90 min in isopropanol and 90 min in paraffin. After processing, the samples were embedded in paraffin blocks, sectioned into 5 μm thick slices, and mounted onto microscope slides. MGT staining was performed on the prepared sections according to a previously established protocol to evaluate collagen organization and structural integrity within the SMAS tissue.^15^

### Statistical Analysis

Data were analyzed using GraphPad Prism 10 (GraphPad Software, San Diego, CA). Parametric values are expressed as mean with standard deviation (SD). To check normal distribution of the variables, the Kolmogorov–Smirnov test was utilized. Parametric data were compared between the PTP-treated samples and controls using paired *t*-test. Results with a *P*-value of <.05 were considered significant. The relative change in biomechanical parameters, such as tissue laxity, modulus, and maximum load, was quantified as a percentage, calculated using the formula: (Experimental − Control)/Control × 100%.

## RESULTS

### Photochemical Passivation Reduces Laxity of SMAS Plication

**Figure 3.**
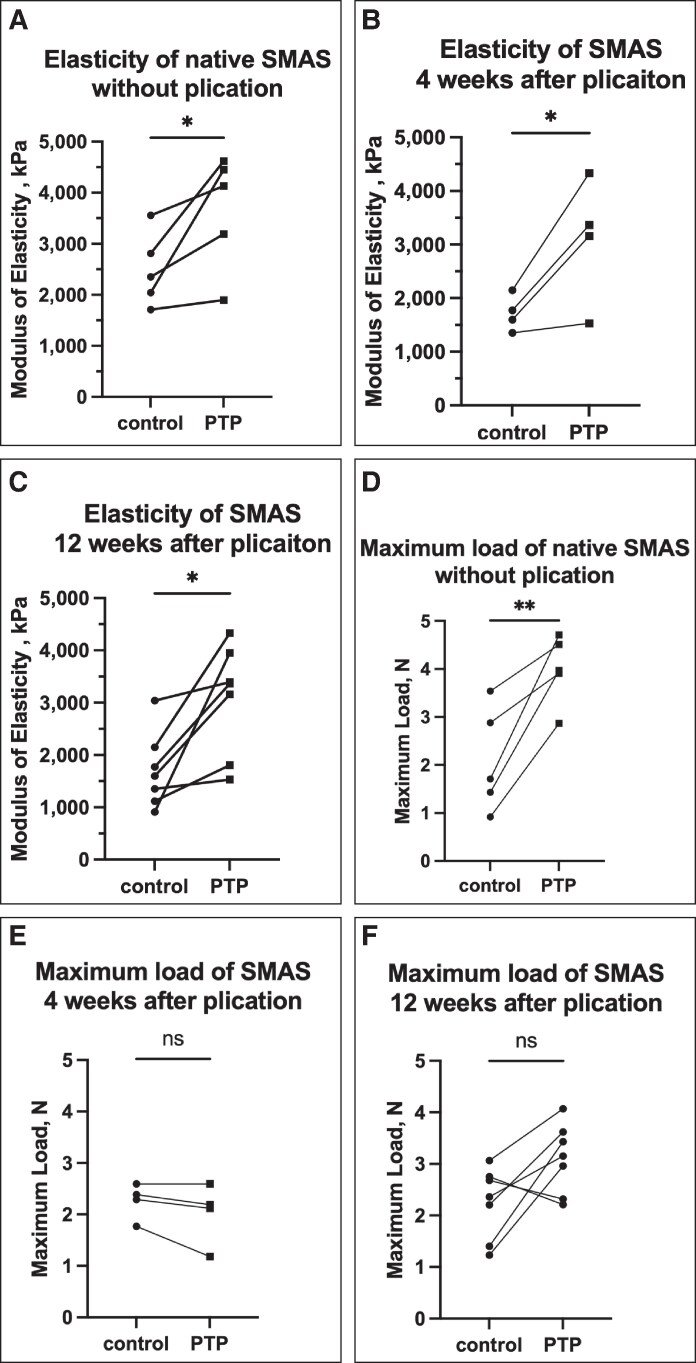
Effects of PTP on biomechanical properties of SMAS after plication in the animal model. (A) The modulus of elasticity was significantly higher in native SMAS immediately after PTP application (*n* = 5; 3659 ± 1128 vs 2495 ± 718 kPa, paired *t*-test, *P* < .05). (B, C) This difference was maintained at both 4 weeks postplication (*n* = 4; 3098 ± 335 vs 1718 ± 1116 kPa, paired *t*-test, *P* < .05) and 12 weeks postplication (*n* = 7; 3077 ± 1043 vs 1706 ± 718 kPa, paired *t*-test, *P* < .05). (D) Maximum load was significantly higher in native SMAS after PTP application (4.0 ± 0.7 vs 2.1 ± 1.1 N, paired *t*-test, *P* < .01), whereas no significant difference was observed at 4 weeks postplication (E) (2.0 ± 0.6 vs 2.3 ± 0.4 N, paired *t*-test, *P* = .15) or 12 weeks postplication (F) (3.1 ± 0.7 vs 2.2 ± 0.7 N, paired *t*-test, *P* = .06). *P < .05; **P < .01; ^ns^*P* > .05. PTP, photochemical tissue passivation; SMAS, superficial musculoaponeurotic system.

The effects of PTP were first evaluated on native, nonoperated SMAS tissue in rats. PTP-treated SMAS tissue (*n* = 5) demonstrated a significantly higher modulus of elasticity compared with control SMAS samples (3659 ± 1128 vs 2495 ± 718 kPa, *P* = .04). Additionally, the maximum load capacity of SMAS was also significantly higher following PTP treatment (4.0 ± 0.7 vs 2.1 ± 1.1 N, *P*≤.009; Figure 3A, D). These findings indicate that PTP enhances the mechanical strength of SMAS, increasing its resistance to deformation under tensile forces and allowing the tissue to withstand greater force before breaking or tearing.

Following multiple pilot trials to establish a reproducible and consistent SMAS plication model, we tested the effect of PTP on the structural integrity and laxity of SMAS plication over time. Four weeks after SMAS plication (*n* = 4), the PTP-treated SMAS exhibited significantly reduced laxity compared with the nontreated side (2.7 ± 0.6 vs 6.4 ± 0.6 mm, *P* = .005). These results were consistent at 12 weeks postsurgery (*n* = 7), with the PTP-treated SMAS maintaining significantly less laxity (3.3 ± 0.5 vs 7.1 ± 1.1 mm, *P* < .0001; Figure 2B, C).

**Figure 4.**
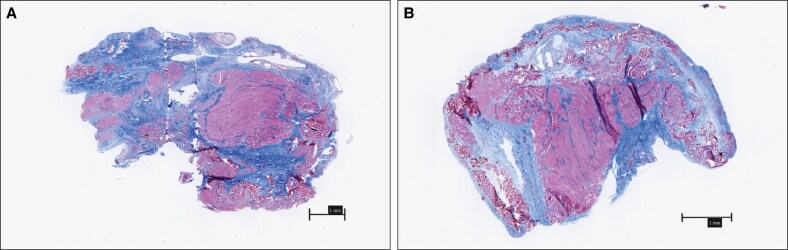
Histological cross-sections of SMAS plication 4 weeks postoperatively. Masson's trichrome staining shows tissue morphology following SMAS plication. (A) In the control (non-PTP) group, the plicated region demonstrates loosely organized collagen (blue) and visible interstitial separation within the fold. (B) In the PTP-treated sample, the plication fold appears more compact with reduced interstitial space and more cohesive alignment of collagen fibers, suggesting improved structural integrity. No residual suture material is visible at this time point. Muscle fibers stain red. Scale bar = 1 mm. PTP, photochemical tissue passivation; SMAS, superficial musculoaponeurotic system.

The modulus of elasticity remained higher in the PTP-treated SMAS at both 4 weeks postplication (3098 ± 335 vs 1718 ± 1116 kPa, *P* = .047) and 12 weeks postplication (3077 ± 1043 vs 1706 ± 718 kPa, *P* = .013; Figure 3B, C). However, the maximum load capacity did not significantly differ between the PTP and control groups at 4 weeks (2.0 ± 0.6 vs 2.3 ± 0.4 N, *P* = .15) or 12 weeks (3.1 ± 0.7 vs 2.2 ± 0.7 N, *P* = .06; Figure 3E, F). Histological analysis of SMAS samples at 4 weeks postplication showed no significant differences in fibrosis, atrophy, or additional collagen accumulation between the PTP-treated and control groups (Figure 4). These findings suggest that PTP enhances the biomechanical stability of SMAS tissue over time without inducing adverse histological changes.

### Human SMAS Exhibits Improved Biomechanical Properties Following Photochemical Passivation

**Figure 5.**
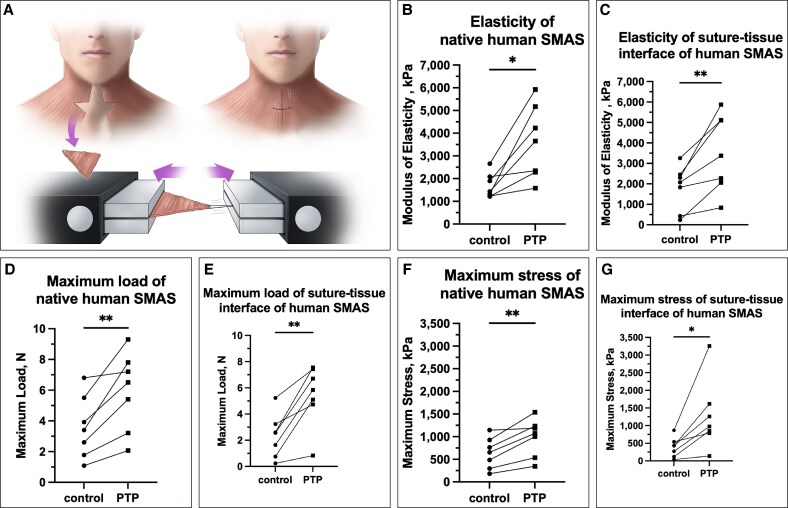
Evaluation of PTP on native human SMAS and suture pull-through analysis. (A) Schematic illustration of tissue harvesting and methodological setup for tensile force testing. (B) Modulus of elasticity was significantly higher in native SMAS after PTP treatment (*n* = 7; 3597 ± 1615 vs 1693 ± 543 kPa, paired *t*-test, *P* < .05) as well as in a simulated surgical setting with a suture placed into the SMAS (C) (3518 ± 1892 vs 1798 ± 1095 kPa, paired *t*-test, *P* < .01). (D) Maximum load was also significantly higher in native SMAS (5.9 ± 2.6 vs 3.6 ± 2.0 N, paired *t*-test, *P* < .01) as well as at the suture–tissue interface (E) (5.5 ± 2.3 vs 2.3 ± 1.7 N, paired *t*-test, *P* < .01). (F) Maximum stress was significantly higher in native SMAS (987 ± 414 vs 637 ± 343 kPa, paired *t*-test, *P* < .01) as well as in the suture–tissue interface group (G) (1269 ± 987 vs 386 ± 278 kPa, paired *t*-test, *P* < .05), indicating improved tissue resistance to mechanical failure and enhanced suture retention strength following PTP treatment. *P < .05; **P < 0.01. PTP, photochemical tissue passivation; SMAS, superficial musculoaponeurotic system.

After evaluating the effects of PTP in the animal model, we conducted an ex vivo human trial (*n* = 7) to assess PTP effects on human SMAS tissue (Figure 5A). The results mirrored those observed in the animal experiments, demonstrating a significant increase in modulus of elasticity (3597 ± 1615 vs 1693 ± 543 kPa, *P* = .01) and maximum load capacity (5.9 ± 2.6 vs 3.6 ± 2.0 N, *P* = .006), indicating enhanced structural integrity and improved resistance to tensile forces. Additionally, maximum stress was significantly higher in the PTP-treated SMAS (987 ± 414 vs 637 ± 343 kPa, *P* = .004), suggesting that PTP strengthens SMAS at the microscopic level by increasing its intrinsic resistance to localized tearing and mechanical failure (Figure 5B, D, F).

To further assess the clinical relevance of PTP, we emulated a surgical setting to test suture retention strength in SMAS tissue. In this model, PTP-treated SMAS exhibited a significantly higher modulus of elasticity (3518 ± 1892 vs 1798 ± 1095 kPa, *P* = .007) and increased maximum load capacity (5.5 ± 2.3 vs 2.3 ± 1.7 N, *P* = .002), indicating enhanced bulk mechanical strength and improved overall tissue durability. Moreover, maximum stress at the suture–tissue interface was significantly higher in the PTP-treated group (1269 ± 987 vs 386 ± 278 kPa, *P* = .002), demonstrating greater resistance to suture pull-through and a lower risk of suture cutting through the tissue (Figure 5C, E, G). These findings suggest that PTP treatment enhances SMAS tissue stability under surgical conditions, reducing the likelihood of suture-related failure.

## DISCUSSION

Facelift procedures remain the most viable and sustainable option for reducing wrinkles and rejuvenating the face.^1^ Although novel techniques have emerged over the decades, little progress has been made in addressing the biomechanical properties of the SMAS itself. In this study, we tested the hypothesis of whether PTP could change the biomechanical properties of the SMAS, enhance its strength, and reduce the risk of suture pull-through failure. Our findings demonstrated improved biomechanical properties of the SMAS in both human and animal models following PTP treatment. This resulted in a 58% improvement in SMAS plication laxity at 4 weeks in the animal model, which was sustained at 54% 12 weeks after surgery. The most striking finding of our study was that PTP increased the localized resistance of human SMAS to suture pull-through by 229%, significantly decreasing the risk of early failure.

The SMAS is an anatomically and biomechanically intriguing structure.^7^ Recent reports have even questioned whether the SMAS exists as a distinct anatomical entity, as its definition remains debated.^6^ Although the first formal description of the SMAS by Mitz and Peyronie in 1976 led to its widespread acceptance as a key surgical layer in facelifts, earlier surgeons unknowingly manipulated the SMAS without naming it.^16^ For instance, Noël recognized that superficial skin tightening alone was insufficient for long-term results, implicitly acknowledging the importance of deeper tissue layers in facial aging and rejuvenation.^17^ Despite the ongoing debate regarding whether the SMAS is a distinct anatomical structure or a surgical concept, similar fascial systems appear in other species.^18,19^ Nonhuman primates and certain mammals exhibit analogous layers resembling the SMAS, though, in most animals, the platysma remains the dominant musculoaponeurotic structure, covering a much larger area of the face and neck.^20,21^ This raises an interesting evolutionary perspective, as it suggests that the SMAS may have developed as a refinement of the platysma, adapting to support the highly segmented and specialized facial musculature in humans.^22^ Although the precise origin of the SMAS remains unclear, its role in facial rejuvenation procedures is undeniable.

Although rodents do not possess a true SMAS, they have an extended platysma covering the entire face.^18^ Given its biomechanical and evolutionary similarities, we established a reproducible SMAS plication model in a small animal using the platysma and fibrotic fascial layers, which resemble human SMAS behavior. Our findings suggest that this model provides a reliable platform to study SMAS plication techniques in facelift surgery (Figure 1). However, the primary limitations of this model include differences in tissue dimensions compared with the human face and inherent viscoelastic differences between rat and human SMAS. Although the biomechanical principle of creating tension on a structurally similar tissue is valid, further refinements are necessary. Although animal models exist for face transplantation and blepharoplasty, no established rhytidectomy (facelift) model exists to the best of our knowledge.^23^ This model may serve as a valuable tool for optimizing and innovating facelift techniques.

Although the established SMAS plication model holds potential for advancing surgical approaches in facelift research, there is no current nonsurgical method to modify the intrinsic properties of the SMAS itself.^24,25^ This technology presents an exciting opportunity for innovation in rhytidectomy techniques. PTP is a promising modality for modifying soft tissue biomechanical properties and has demonstrated significant translational potential in multiple studies.^11,13,14^ This technique is feasible for intraoperative application and could offer a new adjunct to traditional facelift methods. Clinically, numerous facelift techniques exist, including deep-plane and minimally invasive endoscopic approaches. Yet, they all face common limitations such as reoperations, progressive soft tissue laxity, and the inability to retain the SMAS in a stable position over time.^4,26-28^ These issues are largely because of the inherent biomechanical and biological properties of the SMAS, which cannot be permanently altered by surgical techniques alone. Modifying SMAS properties using PTP in a plication model may offer a more sustainable and durable approach to strengthening facelift results. Our data demonstrate improved SMAS retention following plication, suggesting that this technique could enhance facelift longevity, potentially reducing the need for multiple revision procedures and expanding the possibilities for further tissue manipulation and improved outcomes. Moreover, the technique could theoretically be applied in deep-plane, plication, or imbrication approaches, wherever SMAS anchoring is required. Because the method is rapid and nondestructive, it is adaptable to various facelift techniques. Although not tested in this study, a 2-stage procedure—initial PTP followed by delayed SMAS plication—could allow additional tissue remodeling, potentially increasing tensile strength. However, this approach introduces additional patient burden and operative time, warranting further investigation.

Another key finding of this study was that PTP-treated human SMAS demonstrated significantly greater resistance to suture pull-through. In secondary facelifts and revision surgeries, one of the most frequent complications is SMAS fragility, making secure suture placement difficult.^29-31^ The ability to adequately fixate the SMAS in these cases is crucial for a successful outcome. Our findings indicate that PTP increased both the maximum load and maximum stress of the SMAS, which suggests enhanced bulk tissue strength and improved suture retention capabilities. Beyond the clinical application in SMAS plication, PTP could be integrated intraoperatively during facelift surgery after SMAS exposure. Strengthening the SMAS in situ could allow for more secure suture placement, reduced risk of tear-through during plication or fixation, and potentially greater precision during tissue redraping. The PTP procedure can be completed in under 7 min and requires minimal additional resources. RB is cost-effective, and the required laser equipment is already available in many operating rooms. The reinforced tissue could provide significant advantages in secondary facelifts, where weakened and scarred SMAS tissue often compromises surgical results. By improving the resilience of the SMAS, PTP may facilitate more durable fixation and optimize long-term clinical outcomes in revision procedures.

Despite these promising findings, several limitations of our study must be acknowledged. The SMAS plication model in small animals is only an approximation of real-life rhytidectomy, and further studies are needed to evaluate long-term outcomes in human participants. Moreover, the human SMAS samples were treated and tested ex vivo, lacking perfusion, cellular viability, and healing response. Although the biomechanical effects are promising, they may differ in vivo. Moreover, a short follow-up period of 12 weeks also represents a limitation, whereas given that tissue remodeling after facelift surgery can continue for several months or even longer, it is possible that the observed changes in tissue stiffness, swelling, and collagen structure may dissipate or evolve over time. Long-term studies will be necessary to assess the durability of the biomechanical enhancements observed with PTP. The number of human SMAS samples was small (*n* = 7), and additional studies with larger, more diverse cohorts are necessary. Additionally, all human SMAS samples in this study were harvested from the cervical platysma region. Given the known anatomical and biomechanical variability of SMAS across different facial and cervical zones, our findings may not be generalizable to all facelift-relevant areas. The next step in this research would be to translate these findings into larger animal models with more anatomically similar soft tissue properties before advancing to clinical studies in human facelift patients.

## CONCLUSIONS

PTP significantly improves the biomechanical properties of SMAS, enhances suture retention, and reduces postoperative laxity in a validated animal model. These findings lay the foundation for future clinical applications in facelift surgery. Further studies are needed to evaluate safety, timing, and long-term outcomes in human participants.
